# Spontaneous Resolution of Congenital Intrahepatic Portosystemic Shunt

**DOI:** 10.15388/Amed.2024.31.2.14

**Published:** 2024-12-04

**Authors:** Taraprasad Tripathy, Sandeep Behera, Ranjan Patel, Tanmay Dutta, Aditi Kumar, Amit Kumar Satpathy

**Affiliations:** 1 Department of Radiodiagnosis, AIIMS, Bhubaneswar, India; 2 Department of Surgical gastroenterology, AIIMS, Bhubaneswar, India; 3 Department of Pediatrics, AIIMS, Bhubaneswar, India

**Keywords:** congenital portosystemic shunts, embolization, Abernathy malformation, Raktažodžiai: įgimtas portosisteminis šuntas, embolizacija, *Abernathy* malformacija

## Abstract

Congenital portosystemic shunts (CPSS) are a rare type of congenital abnormality. It results from abnormal embryonic development by the fourth week of fetal life. Congenital portosystemic shunts are believed to signify persistent communication between the portal and vitelline venous systems. Both extrahepatic and intrahepatic shunts are possible. They may develop on their own or in association with other congenital abnormalities. With regard to shunt type and size, symptoms vary widely. The anatomy of the shunt and associated abnormalities can be assessed by computed tomography and magnetic resonance angiography. The treatment plan is based on the type of shunt, its location and level of function, the patient’s age, and the severity of their symptoms.

## Abstract (it)

įgimtas portosisteminis šuntas (angl. *Congenital portosystemic shunts*, CPSS) yra reta įgimta anomalija. Ji atsiranda dėl netaisyklingo embriono vystymosi iki ketvirtos vaisiaus gyvenimo savaitės. Manoma, kad įgimti portosisteminiai šuntai reiškia nuolatinį portalinės ir stiklakūnio venų sistemos ryšį. Galimi tiek ekstrahepatiniai, tiek intrahepatiniai šuntai. Jie gali išsivystyti atskirai arba kartu su kitomis įgimtomis anomalijomis. Atsižvelgiant į šunto tipą ir dydį, simptomai labai skiriasi. Šunto anatomiją ir susijusias anomalijas galima įvertinti kompiuterinės tomografijos ir magnetinio rezonanso angiografijos metodais. Gydymo planas sudaromas atsižvelgiant į šunto tipą, jo vietą ir funkcijos lygį, paciento amžių ir simptomų sunkumą.

## Introduction

Congenital portosystemic venous shunts (CPSS) are uncommon vascular malformations that result from faulty fetal vasculature development or involution [1]. They enable intestinal blood to enter the systemic circulation without going via the liver, which has many long-term effects and consequences. In contrast to secondary portosystemic shunts in the presence of liver cirrhosis or portal vein obstruction, ascites, and portal hypertension are not often characteristics of CPSS. The two types of CPSS are intrahepatic and extrahepatic shunts [2]. Extrahepatic shunts are also known as Abernethy malformations after John Abernethy, who first reported this condition [3]. The pathogenesis and treatments for the two types are different despite their clinical symptoms being similar.

## Case presentation

A 3-month-old male child presented to paediatric OPD with fever, dyspnoea, irritability, and difficulty feeding for approximately one month. On examination, pallor and icterus were present without any cyanosis or clubbing. No abnormal antenatal history was noted. The child was born at term through normal vaginal delivery. There was no history of perinatal asphyxia or neonatal jaundice present. On further biochemical investigation, there was increased bilirubin, Aspartate aminotransferase (449 U/L), Alanine aminotransferase (185 U/L), hyperglycaemia, hyperammonaemia (56 mmol/L), and serum bile acid (21µmoles/L). An ultrasound of the abdomen was advised to look for hepatic pathology in view of hyperbilirubinemia.

**Figure 1:**
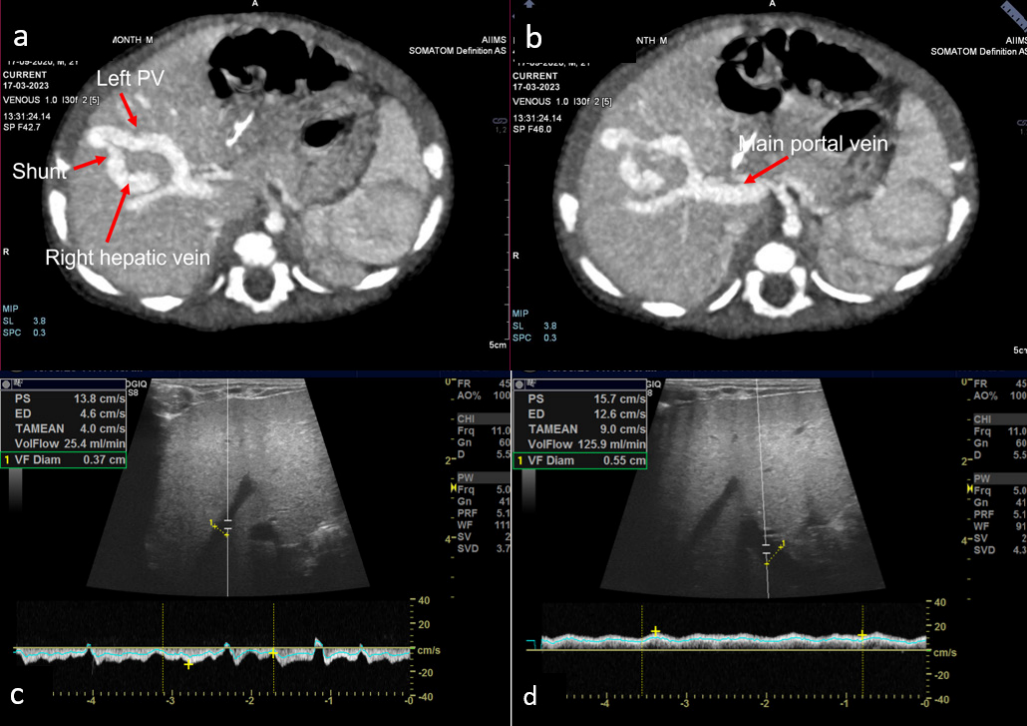
a) USG shows anechoic tubular structure communicating between left portal vein and right hepatic vein. b) On color Doppler there is communication between hepatic vein and portal vein which shows color flow with aliasing. c) Calculation of porto-venous shunt ratio is shown – ~88% in present case.

**Figure 2:**
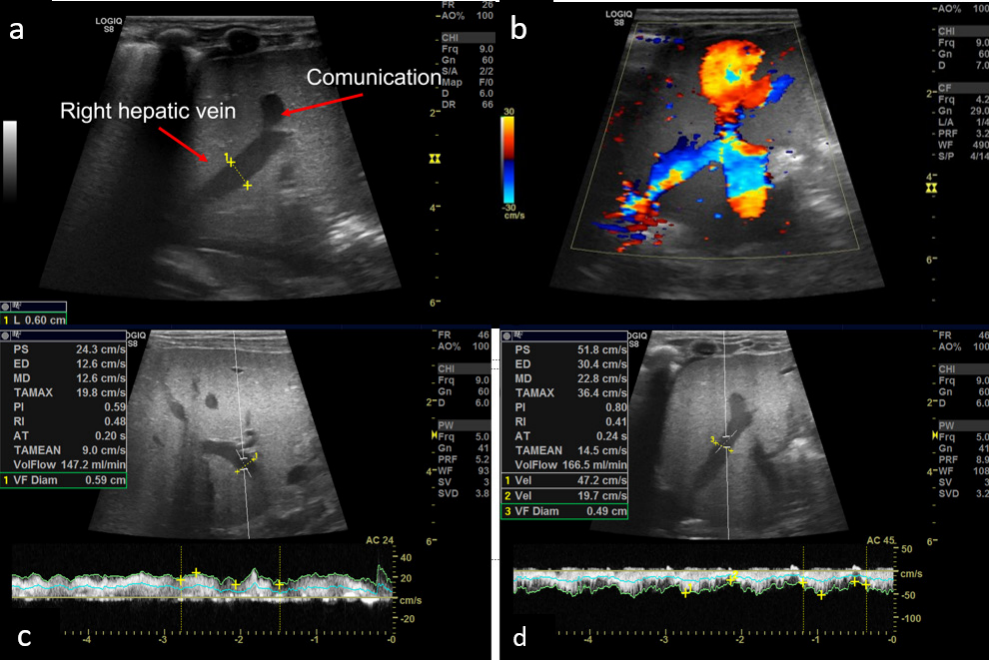
a) and b). CECT porto-venous phase MIP images show communication between branch portal vein and right hepatic vein – suggestive of type 2 congenital portosystemic shunt. b) Follow-up USG after 2 months of presentation shows resolution of shunt. Porto-venous shunt ratio is ~20.1%. (MIP: Maximum intensity projection)

The ultrasound of the abdomen showed an anechoic tubular structure with a maximum diameter of 6 mm, communicating between the branch of left portal vein and the right hepatic vein in periphery of liver. On the color Doppler, the shunt between the hepatic vein and the portal vein, showed color flow and aliasing (figure 1 a and b). Flow dynamics and Doppler parameters were obtained. Porto-venous shunt ratio refers to ratio of blood flow volume through the shunt and through the portal vein from which the shunt arises, was calculated and came out to be approximately 88% (figure 1 c and d). There is hepato-petal flow in main as well as right and left portal veins. No evidence of hepato-fugal flow noted. Liver echotexture was normal; no other space-occupying lesion was noted in the liver. No evidence of any free fluid or splenomegaly was noted. Other solid organs are within normal limits. Echocardiography was unremarkable. Triple-phase contrast-enhanced MDCT (SOMATON DEFINITION FLASH, SIEMENS) was done to further characterize the communication and find any associated congenital anomaly. CECT shows communication between the branch of left main portal vein and the right hepatic vein, which is opacified in the porto-venous phase (figure 2 a and b). This is suggestive of a type 2 congenital intrahepatic portosystemic shunt (according to the classification by Park et al.). No other significant abnormality was present in CT.

Despite having a high shunt ratio, which is usually an indication of definite management, the child was managed conservatively in view of inadequate weight and neonatal cholestasis. Also, symptoms were controlled by conservative management. After symptomatic management, the child became stable and discharged. The child was kept on follow-ups, and danger signs were explained to the parents. On a two-month follow-up, an ultrasound was done to look for shunt function and shunt ratio.

The Doppler showed normal flow and waveform in the right hepatic vein. The anechoic shunt was not visible on gray scale. Peak systolic velocity and volume flow were also reduced compared to ultrasound done two months prior. Porto-venous shunt ratio decreased to a level of 20.1% (figure 2 c and d). It confirms the spontaneous resolution of the shunt. The child was asymptomatic at that time. Further, the child was kept in regular follow-up.

## Discussion

Congenital intrahepatic portosystemic shunt is one of the rare congenital abnormalities. It is classified into four types by Park et al., which characterizes them as communications > 1 mm in diameter between the intrahepatic portal vein and the hepatic or perihepatic veins [2]. It is classified into subtypes based on the location of the shunt, its number, and shunt characteristics (table 1).

**Table 1:** Classification of congenital intrahepatic portosystemic shunts.

Type 1: Single large vessel connecting the right portal vein to the inferior vena cava
Type 2: Localized, peripheral shunt with one or more communications inside a single hepatic segment
Type 3: Peripheral portal and hepatic veins connected through an aneurysmal channel
Type 4: Multiple communications between the peripheral portal and hepatic veins in multiple segments

The first two varieties are the most prevalent, and show male preponderance. Although it travels through the ligamentum venosum, a patent ductus venosus is always referred to as an intrahepatic shunt type 5 since it arises from the left portal vein.

Patients with CPSS exhibit a wide range of symptoms and potentially life-long consequences, while accidentally identified asymptomatic cases are not unusual. The most notable side effects of chronic portosystemic shunting include hepatic encephalopathy, hepatopulmonary syndrome, thrombocytopenia, hyperammonaemia, and pulmonary hypertension, which are more frequently seen in children [4]. Rectal bleeding, though uncommon, can be seen in a few patients with an extrahepatic malformation [5].

In the absence of hypoxia or other evident maternal illnesses and/or chromosomal abnormalities, changes in fetal venous circulation from shunting may cause decreased liver perfusion and symptoms of intrauterine growth restriction. Galactosemia and neonatal cholestasis are possible conditions, but they should be distinguished from other congenital anomalies, including biliary atresia and metabolic conditions that might coexist.

Pancreatitis (related to morphological narrowing of the pancreaticobiliary junction), vaginal bleeding, and symptoms of the lower urinary tract (lithiasis, haematuria) have all been linked to CPSS sequelae. Insulin resistance brought on by the changed hepatic hemodynamic, which leads to hyperandrogenism (in a way similar to liver cirrhosis), is also a possibility. Additionally, these patients are more likely to develop benign regenerative liver lesions, primarily extensive lesions that resemble nodular hyperplasia, although hepatocellular adenomas have also been seen. The underlying congenital vascular abnormality is hypothesized to cause increased arterial perfusion, which triggers a hyperplasic hepatocellular response that leads to FNH [6].

A portosystemic venous fistula should be considered a differential diagnosis in a patient with recurrent hepatic encephalopathy who is either early cirrhotic or noncirrhotic [6]. Treatment generally depends upon the age at presentation, the severity of symptoms, the presence of neonatal cholestasis, the shunt ratio, and associated congenital abnormalities. Patients are usually made hemodynamically stable first. In the case of small shunt (porto-venous shunt ratio <60 %), patients are generally managed symptomatically as they have a high chance of spontaneous closure. In patients with a higher shunt ratio (>80%), definite treatment is advised.

According to previous studies, the spontaneous closure rate of CPSS varies greatly between 5 and 52%, depending on multiple factors, mainly the type of shunt and shunt size [4]. A study by Paganelli M et al. found that the shunt type and neonatal cholestasis highly predict spontaneous shunt closure before the 24th month of life. It may be possible to prevent the need for early shunt closure and the risks associated with surgical or endovascular procedures in newborns with neonatal cholestasis and uncomplicated CPSS by following “wait and see” policy [7].

Definite management can be surgical or endovascular. With the advent of minimally invasive endovascular therapy, surgical treatment is now in less use. Embolization is generally achieved through micro coils. However, some cases were also managed with n-butyl cyanoacrylate. Shunt size and morphology should be taken into consideration when choosing an embolic agent from the variety of options available, such as coils, Gelfoam® particles, and polyvinyl alcohol [8]. Gupta et al. have reported successful embolization of congenital intrahepatic shunt with n-butyl cyanoacrylate in a 14-month-old child [9]. In another study, Lee et al. reported embolization using the Amplatz vascular plug II [8]. In severe cases, surgical excision of the shunt and a liver transplant may be required.

## Conclusion

Congenital portosystemic shunts are rare entities with a wide range of clinical presentations. A high degree of clinical suspicion is essential to make a definite diagnosis. USG acts as a diagnostic screening tool and rules out other causes with similar clinical presentation. Cross-sectional imaging is used for accurate delineation as well as for treatment planning. Therapeutic decision making depends on multiple factors like type of shunt, shunt size and neonatal cholestasis. It may be possible to prevent the need for early shunt closure and the risks associated with surgical or endovascular procedures in newborns with cholestasis and smaller/peripheral intrahepatic shunts.

## Abbreviations

CPSS – Congenital Porto-Systemic Shunts

OPD – Out Patient Department

USG – Ultrasonography

MDCT – Multi Detector Computed Tomography

CECT – Contrast Enhanced Computed Tomography

CT – Computed Tomography

FNH – Focal Nodular Hyperplasia
